# Lectures on the Diseases of Infancy and Childhood

**Published:** 1854-07

**Authors:** 


For sale by MILLER, ORTON & MULLIGAN. H.

---

ART. VII.—*Lectures on the Diseases of Infancy and Childhood.* By CHARLES WEST, M. D., F. R. C. P., etc., etc. Second American from the second and enlarged English edition. Philadelphia: Blanchard and Lea. 1854.

We are glad to see a second edition of this work. We have long regarded it as the most truly scientific and practical book on diseases of children, which has yet appeared in this country.

The work is not so full as Condie's, neither does it enumerate so large a number of diseases. But it has the merit of exhausting the subjects of which it treats; of presenting all of the most valuable and important facts, in a condensed, and statistical style. The style is very similar to that of Watson's lectures, and like them readable and interesting.

We have frequently had occasion, in noticing works on the diseases of childhood, to regret that the treatment was not more carefully adapted to the tender age, and impressible systems, of the little patients. All authors are somewhat liable to this charge. The pages of our standard authors are filled with heroic prescriptions, such as no prudent practitioner finds necessary. Blood-letting, cupping, leeching, calomel, and antimony, are as freely recommended for the feeble and susceptible child, as for the robust adult.

Those ideas of active antiphlogistic treatment, which are so rapidly passing from the pages of modern works as a general practice, are still perseveringly retained in treatises on infantile disease. This is, to some extent, the case with West, but he is not so open to the charge as many others. His varied prescriptions indicate a corresponding variance in the disease under consideration, and a nice adaptation of remedies to different morbid conditions.

Among the subjects which Dr. West has treated with a peculiar skill, derived from a long attendance on a hospital for sick children, is that of hydrocephalus. He draws a line between those *hydrocephaloid* diseases which occurred in prolonged diarrhœa, or other mucous disease, and those genuine forms of local cerebral disease, which are *sui-generis.* This distinction, always difficult, and frequently impossible, is still very important. Thus, it sometimes happens, that a pneunomia is ushered in by cerebral symptoms, so severe as to mask the real condition of the patient. So, too, in the sympathetic cerebral disease which attends prolonged diarrhœa. The practitioner who directs his treatment to the head, will find but little benefit from a treatment, which disregards the disease, and follows only the prominent symptoms.

On the subject of tubercular meningitis our author is particularly full and accurate. We notice in one case he found a single, cretaceous tubercle, of which the albuminous part had undergone absorption. The child, in this case, died of some intercurrent disease, in no way dependent on the cerebral lesion. We have reason to believe that a case now in progress, and which we have seen in consultation, is undergoing the same recovery. That such a recovery is possible we can readily believe. It is probable that, as many cases of simple hydrocephalus are mistaken for the tubercular form, so also, it may sometimes happen that tubercle may undergo absorption, and the true pathology of the case pass unrecognized. Our present prognosis in the case alluded to, is, that the patient will recover as an idiot, possibly from the pressure of cretaceous deposit upon the cerebral mass.

We cannot part with West on Diseases of Children, without calling attention to the condition which he calls collapse of lung, as differing from true lobular pneumonia. The former of them has been usually considered as identical with the latter, the solidification found being thought to be red hepatization. A very important distinction should be made here.

Collapse of lung is a condition supervening upon bronchitis, or sometimes from a deficiency of inspiratory power. In the former case a bronchial tube becomes plugged by mucus, or by swelling; thus closing the portion of lung varying in size from that of a pea, to that of an almond or even larger. Many of these solidified portions may occur in the same lung. It is found in post-mortem examinations, that those solidifications may be expanded by the blow-pipe, thus proving them to be, not true hepatization, but a mere compression, or *collapse* of lung. The symptoms do not vary much from lobular pneumonia, which is merely a continuation of bronchitic inflammation to the air vesicles. But it will be found in collapse of lung, that, though the physical signs are the same, (with the exception of the crepitant râle) the rational signs differ materially. The fever is less persistent, the cough ordinarily dry and the sputa not rusty, and a process of emaciation is set up, with enfeebled digestion and strength, and, as we have noticed, an unusual fretfulness of temper. Of course the treatment should differ. Lobular pneumonia requires a more or less antiphlogistic management, while collapse of lung requires a decidedly stimulant and tonic treatment. Carb-ammon., quinine, wine, and stimulating liniments, are of the greatest service, and under them we have seen very hopeless cases of its chronic form recover. Sometimes it occurs in so sudden a manner, and over so large an extent of lung, as to prove immediately fatal. It is in this manner that many cases of bronchitis terminate.

